# Benthic primary production and respiration of shallow rocky habitats: a case study from South Bay (Doumer Island, Western Antarctic Peninsula)

**DOI:** 10.1007/s00300-019-02533-0

**Published:** 2019-07-16

**Authors:** Lorenzo Rovelli, Karl M. Attard, César A. Cárdenas, Ronnie N. Glud

**Affiliations:** 1 Nordcee, Department of Biology, University of Southern Denmark, 5230 Odense M, Denmark; 2 Tvärminne Zoological Station, University of Helsinki, 10900 Hanko, Finland; 3 Departamento Científico, Instituto Antártico Chileno, 6200965 Punta Arenas, Chile; 4 Department of Ocean and Environmental Sciences, Tokyo University of Marine Science and Technology, Tokyo, 108-8477 Japan; 5 Present Address: Institute for Environmental Sciences, University of Koblenz-Landau, Landau, Germany

**Keywords:** Aquatic eddy covariance, Community oxygen exchange, Doumer Island, Base Yelcho

## Abstract

Rocky benthic communities are common in Antarctic coastal habitats; yet little is known about their carbon turnover rates. Here, we performed a broad survey of shallow ( < 65 m depth) rocky ice-scoured habitats of South Bay (Doumer Island, Western Antarctic Peninsula), combining (i) biodiversity assessments from benthic imaging, and (ii) in situ benthic dissolved oxygen (O_2_) exchange rates quantified by the aquatic eddy covariance technique. The 18 study sites revealed a gradual transition from macroalgae and coralline-dominated communities at ice-impacted depths (15–25 m; zone I) to large suspension feeders (e.g., sponges, bivalves) at depth zone II (25–40 m) and extensive suspension feeders at the deepest study location (zone III; 40–65 m). Gross primary production (GPP) in zone I was up to 70 mmol O_2_ m^−2^ d^−1^ and dark ecosystem respiration (ER) ranged from 15 to 90 mmol m^−2^ d^−1^. Zone II exhibited reduced GPP (average 1.1 mmol m^−2^ d^−1^) and ER rates from 6 to 36 mmol m^−2^ d^−1^, whereas aphotic zone III exhibited ER between 1 and 6 mmol m^−2^ d^−1^. Benthic ER exceeded GPP at all study sites, with daily net ecosystem metabolism (NEM) ranging from − 22 mmol m^−2^ d^−1^ at the shallow sites to − 4 mmol m^−2^ d^−1^ at 60 m. Similar NEM dynamics have been observed for hard-substrate Arctic habitats at comparable depths. Despite relatively high GPP during summer, coastal rocky habitats appear net heterotrophic. This is likely due to active drawdown of organic material by suspension-feeding communities that are key for biogeochemical and ecological functioning of high-latitude coastal ecosystems.

**Electronic supplementary material:**

The online version of this article (10.1007/s00300-019-02533-0) contains supplementary material, which is available to authorized users.

## Introduction

Shallow coastal areas of Antarctica cover ~ 18,400 km^2^ (0–50 m depth; Barnes 2017). For most of the year, this region is covered by sea ice with a persistent snow layer limiting light availability of the under-ice environment (Arndt et al. 2017). However, in summer most sea ice is melted, and the coastal Antarctic waters become highly productive, sustaining a remarkable and diverse marine ecosystem which spans from microscopic algae and invertebrates to penguins, seabirds, and large marine mammals (see Convey et al. 2014). Despite year-round cold water temperatures and strong seasonality, coastal benthic communities are characterized by high species richness (see Clarke and Johnston 2003; Griffiths and Waller 2016). In the Western Antarctic Peninsula (WAP), for instance, large macroalgae are widely distributed (e.g., Amsler et al. 1995; Klöser et al. 1996; Quartino et al. 2001), as are dense benthic faunal assemblages of, e.g., echinoderms (White et al. 2012), gastropods (Amsler et al. 2015), ascidians (Lagger et al. 2018), and sponges (Cárdenas et al. 2016).

The distribution of benthic communities is driven by the nature of the seabed, the bathymetry, and the seasonal variations in physical drivers (Nonato et al. 2000; Cummings et al. 2006) as well as biological factors such as recruitment and predation (Dayton et al. 1974; Smale 2008). These include (i) extent and dynamics of the ice cover, which modulates light availability, stratification, pelagic primary production, and thus the supply of organic material and nutrition to the benthic compartment, and (ii) ice disturbances such as ice scouring, which annually can impact ~ 30% of the coastal Antarctic seafloor at depths < 25 m (Barnes 2017). The resulting Antarctic coastal benthic habitats show considerable change in the distribution and composition of algae and benthic fauna along a bathymetric gradient (Brouwer et al. 1995; Nonato et al. 2000) and an extensive spatial heterogeneity (Gutt 2000; Teixidó et al. 2002; Smale 2008).

Previous investigations in the Arctic have documented the important role of shallow benthic habitats for primary production and respiration processes in the coastal zone (Glud et al. 2009; Attard et al. 2014). While data in the Arctic remain scarce, Antarctic research on the mentioned topics is even harder to come by, with some areas even lacking basic information on, e.g., the bathymetry (e.g., Gattuso et al. 2006). The existing studies have mainly targeted soft-sediment communities by applying sediment incubations (Nedwell et al. 1993; Nedwell and Walker 1995; Shim et al. 2011; Hoffmann et al. 2018; Braeckman et al. 2019) and O_2_ microprofile measurements (McMinn et al. 2010; Hoffmann et al. 2018). However, hard substrate, i.e., rocky habitats are a dominant feature in Antarctic coastal waters, that cannot be targeted by traditional measuring approaches and they have thus been overlooked or ignored for assessing coastal element cycling (e.g., Glud et al. 2010).

The introduction of the aquatic eddy covariance (AEC) technique (Berg et al. 2003) has made it possible to quantify in situ vertical fluxes of dissolved oxygen (O_2_) on complex marine benthic habitats occurring on hard substrates such as coral reefs (Long et al. 2013; Rovelli et al. 2015), permeable sand (Berg et al. 2013; McGinnis et al. 2014), rocky outcrops, pebbles, and coralline communities (Glud et al. 2010; Attard et al. 2014, 2016). The main advantage of the AEC technique over traditional methods is that the AEC can non-invasively quantify the benthic O_2_ exchange of complex benthic habitats integrating measurements over larger (10–100 m^2^) seafloor surface areas and not altering the local hydrodynamics, food, and light availability. The AEC technique thus represents a valuable tool for assessing the benthic carbon turnover of heterogeneous coastal benthic communities in polar settings.

Here, we present the first quantification of O_2_ flux dynamics for shallow Antarctic benthic communities on hard substrate using the non-invasive AEC technique. The acquired O_2_ fluxes were used to derive key metabolic parameters such as gross primary production (GPP), ecosystem respiration (ER), and net ecosystem metabolism (NEM) along a depth transect. Combined with biodiversity assessments from concurrent benthic imaging surveys, the data are used to (i) describe the heterogeneity and bathymetrical changes of a typical Antarctic benthic community and its integrated metabolic parameters, and (ii) relate the inherent variability of those habitats to key physical drivers. The results provide a broader, and much needed insight into the ecosystem functioning of hard-substrate coastal communities in polar regions.

## Materials and methods

### Study site

**Fig. 1:**
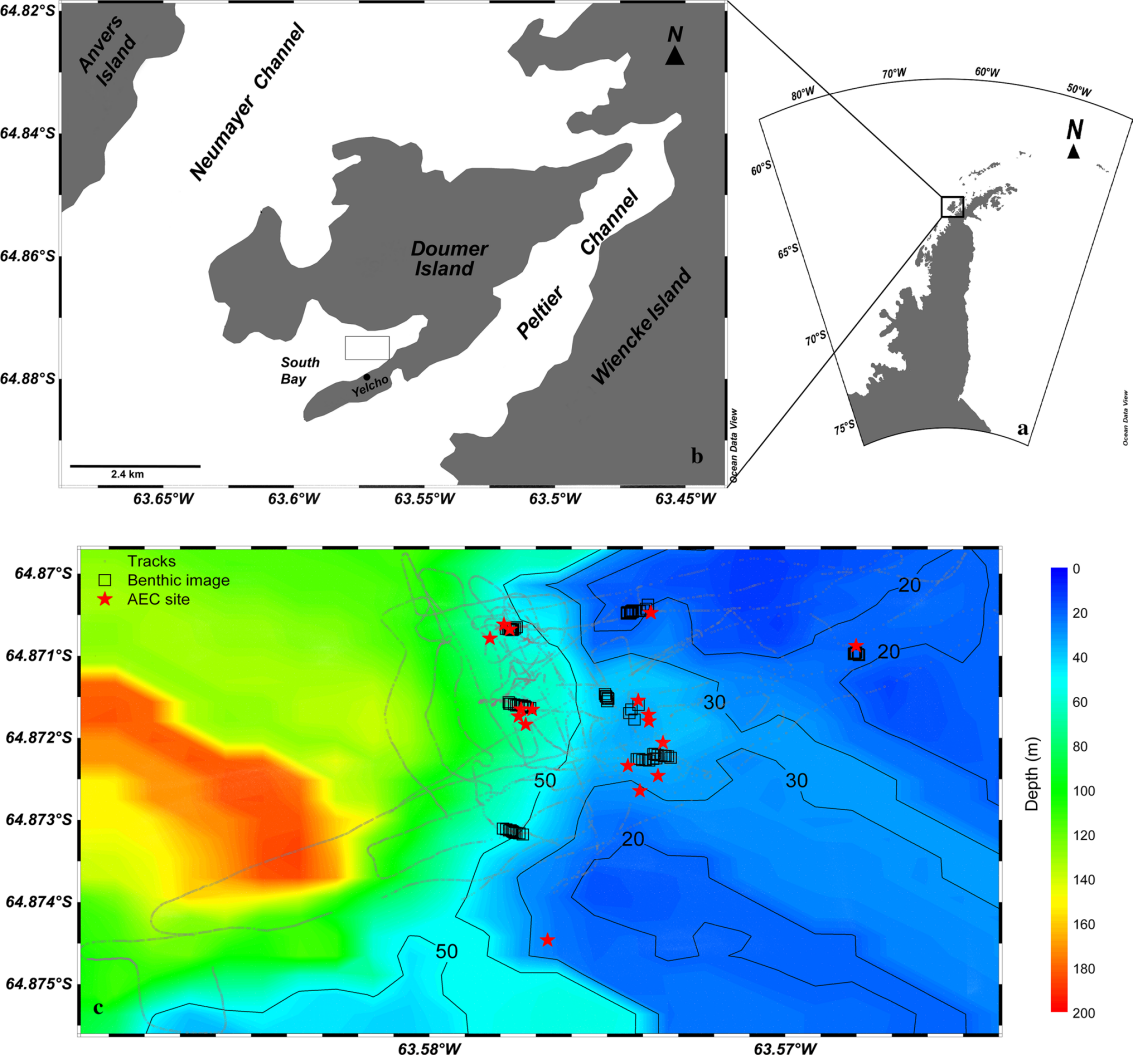
Location of South Bay within the Western Antarctic Peninsula (**a**, **b**) and updated bathymetric map from the 2017 expedition survey (**c**). Open squares indicate the benthic images taken during the biodiversity survey, while deployment sites of the aquatic eddy covariance (AEC) systems are depicted as red stars. Ship tracks are shown as gray dots

The study was performed in South Bay (Doumer Island; 64°52′32″S, 63°35′02″W), WAP, during the Austral summer (Jan–Feb) 2017 (Fig. 1). The bay covers an area of ~ 2.3 km^2^, with water depths ranging from < 30 m at the North-East end of the bay, to 60–90 m at the mouth of the bay (South-West end), and to up to 222 m in the central region. The fieldwork was carried out from the Chilean Antarctic research station Yelcho, located on the South shore of the bay. Most of the research activities at the station have been performed in the 1970–1980s and until the late ‘90s under the administration of the Chilean Antarctic Institute (INACH). This work has provided pioneering surveys of the ecology and benthic biodiversity in the area (e.g., Moreno et al. 1977; Moreno 1980; Moreno and Osorio 1980; Zamorano 1983; Zamorano et al. 1986).

These previous studies, mostly performed by divers, revealed highly heterogeneous benthic habitats with a distinct bathymetric change (Zamorano 1983; Cárdenas et al. 2016). Benthic habitats in shallow areas ( < 18 m depth) are mostly characterized by rocky steep slopes. Within these areas, primary producers are dominated by red algae (e.g., *Lithothamnium* sp*.*, *Gigartina skottsbergii*) within the upper region (0–7 m depth) and by brown algae (e.g., *Desmarestia* spp.*, Himantothallus grandifolius*) down to 25 m depth. Benthic fauna within the upper region is dominated by the Antarctic limpet (*Nacella concinna*), and to a lesser extent, by sea stars (e.g., *Odontaster validus*) and crustaceans. At intermediary depths (15–25 m) sand-mud plains host benthic infauna such as bivalves (e.g., *Laternula elliptica, Aequiyoldia eightsii*), polychaetes (*Maldane sarsi*) and sea urchins (e.g., *Abatus cavernosus*). Below 25 m depth, the main habitats include rocky bottoms with accumulated gravel and thin deposits of soft sediment. These habitats are characterized by the highest faunal species richness, dominated by sponges (e.g., *Mycale acerata*, *Dendrilla antarctica*, and *Rosella nuda*) and ascidians (e.g., *Cnemidocarpa verrucosa*).

These early surveys were limited to 30 m depth, but it has been presumed that communities at these depths extended further into deeper waters. Due to the termination of station operations in 1998, however, the South Bay coastal ecosystem has not been further investigated. The re-opening and modernization of the station in 2015 has offered the opportunity to revisit South Bay, and expand the pioneering surveys using both traditional and novel scientific approaches. This has shed light on the intricate dynamics within the bay’s ecosystem, from background marine and meteorological measurements, e.g., water temperature, wind direction and magnitude, and water column productivity (Cárdenas et al. 2018a; Villegas et al. 2018), to the role of water column stability for pelagic primary production (Höfer et al. 2019), to revised assessments of sponge species’ richness in macroalgae-dominated shallow habitats and prokaryotic communities associated with sponges (Cárdenas et al. 2016, 2018b).

### Spatial survey

#### Bathymetry

In this study, existing coarse bathymetric surveys of the area (e.g., Zamorano 1983) were expanded by high-resolution mapping using an echoMAP Chirp 52dv echo sounder (Garmin International Inc., Olathe, USA) equipped with a DownVü transducer (model GT20, 77, and 200 kHz). The improved bathymetric maps were used to identify and better constrain potential areas for AEC deployments (see below).

#### Benthic imaging

Visual surveys of benthic features and biodiversity were performed with a Sony RX100 III camera equipped with an underwater housing (Sea&Sea, Tokyo, Japan) and two Sola Video 2100 lights (Light & Motion, Marina, USA) that were mounted on a metal frame. The surveys were divided into 11 transects of 10–30 m in length, during which the frame was lowered to 1–2 m height above the bottom and, after a short period, hopped repeatedly at the seabed while the boat drifted. Each transect delivered up to 12 benthic images that were extracted from video footage at each landing using the VLC (Videolan, Paris, France) software. The camera field of view was determined using metered poles along the frame’s *x* and *y* axis. The usable spatial coverage of the calibrated images, excluding optical distortion at the frame corners, was 41 × 73 cm (0.3 m^2^).

### Biodiversity

Biodiversity analyses were performed visually on each high-resolution image to identify key algae and faunal taxa as well as type of benthic substrate. Observed algae were categorized into brown algae, green algae, red algae, corallines, as well as filamentous algae and benthic microalgae.

Benthic fauna groups included Bryozoa, Porifera, Holothuroidea, Hydrozoa, Ascidiacea, Anthozoa, Bivalvia, and Polychaeta. The relative abundance of each taxon was reported as habitat coverage, i.e., percentage of the image surface. The relative coverage of benthic categories was estimated by superimposing a grid of 100 points onto each high-resolution image using the software CPCe (Coral Point Count with Excel extensions) v3.5 (Kohler and Gill 2006). For motile fauna, areal abundances were also estimated by counting the number of individuals on each picture. Exposed substrate categories included bare sediment, sand, gravel, and small to large rocks not colonized by macrobiota. Hard substrate, ranging from gravel to large boulders, was found to be the dominant within the investigated areas of South Bay, whereas soft substrate was typically limited to occasional thin surficial layers of deposited sediment on a rocky base. Patchy soft-sediment habitats have been reported at depths from 15 to 22 m toward the Eastern shore of the bay (see Zamorano 1983) but these were outside of the areas investigated within this study.

### Aquatic eddy covariance measurements

#### Site selection

Suitable areas for AEC deployments were identified based on the following selection criteria: (i) the occurrence of a well-defined flat area ( < 2 m elevation difference) over an area of 50 m × 50 m, and (ii) absence of sharp topographic features (i.e., vertical drops, channels, depressions) in the vicinity of the targeted deployment spot. Prior to AEC deployments, the selected sites were inspected by imaging surveys to identify potential deployment hazards and large rocks that could lead to a tilted positioning of the AEC frame. The procedure identified a number of transects within a depth range of 15–60 m with established benthic communities around the AEC deployment sites.

#### Instrument setup

Benthic O_2_ fluxes were obtained using two AEC systems. The systems were similar to the original design by Berg and Huettel (2008) and consisted of an acoustic Doppler velocimeter (ADV; Vector, Nortek), submersible amplifiers (see McGinnis et al. 2011), and Clark-type O_2_ microelectrodes (Revsbech 1989), all mounted on a stainless-steel tripod frame. Each microelectrode had a tip of ~ 50 µm, a (90%) response time ≤ 0.5 s, and low stirring sensitivity (< 0.5%; see Gundersen et al. 1998). Recorded 64 Hz ADV datasets included three-dimensional flow velocity and O_2_ microsensor signals and complementary information on sampling distance from the seabed, flow direction, acoustic signal strength, and hydrostatic pressure. The ADV was mounted downward-looking at a measurement height (*h*) of ~ 25–30 cm above the seabed depending on the seafloor topography at each site. The O_2_ microelectrodes were positioned 0.5 cm off the 2.16 cm^3^ ADV sampling volume and at a 60° angle to provide robust data cross-correlation (e.g., Donis et al. 2015) and facilitate time shift corrections (see “Data processing”). A small O_2_ optode and conductivity-temperature loggers (Onset Computer Corporation, Bourne, USA) were mounted on each frame to enable in situ calibrations of the AEC microelectrodes. Light availability was quantified as photosynthetically active radiation (PAR) using a frame-mounted logger (RBR Solo; RBR Ltd., Ottawa, Canada) equipped with a 2π underwater PAR sensor (Li-Cor, Lincoln, USA). The AEC instrument was lowered to the seafloor by hand from a small research vessel, and a U-mooring was laid out for instrument retrieval. Individual deployments typically lasted 20–80 h.

#### Data processing

The collected ADV datasets were processed following established protocols (e.g., Attard et al. 2014; Rovelli et al. 2017). In brief, time series were averaged from 64 to 8 Hz to reduce instrument noise levels and reduce data size for more efficient data analyses. During the averaging procedure, ADV velocity data with beam correlations < 50% and signal-to-noise ratios (SNR) < 5 were flagged and subsequently replaced by linear interpolation. Averaged O_2_ and velocity time series were despiked using the Matlab despiking toolbox of Goring and Nikora (2002). To account for the structural complexity of the observed benthic habitats and their impact on the local flow field, a planar-fit rotation was applied to the ADV velocity datasets (Lorke et al. 2013). The time-averaged turbulent benthic O_2_ fluxes (*F*) were obtained from fluctuations of vertical velocity ($w^{\prime}$) and O_2_ concentration ($C^{\prime}$) as $F=\overline{w^{\prime}C^{\prime}}$ (Berg et al. 2003). Fluctuations were quantified based on Reynolds decomposition as ${w}^{\prime}=w-\overline{w}$ and ${C}^{\prime}=C-{\overline{C}}$ with *w* and *C* being the measured vertical velocities and O_2_ concentrations, respectively, and ${\overline{w}}$ and ${\overline{C}}$ the time-averaged values. The actual decomposition was performed by linear detrending. Dataset was time-shifted to account for the distance between ADV sampling volume and sensing O_2_ microelectrode and the response time of the microelectrodes (Donis et al. 2015) using the Fortran program suite Sulfide-Oxygen-Heat Flux Eddy Analysis version 2.0 (see McGinnis et al. 2014). The time interval (window) for estimating turbulent fluctuations was inferred from increasing window sizes for the determination of O_2_ fluxes and shear velocity (u_*_) (McGinnis et al. 2008; McPhee 2008; Attard et al. 2014). Mean u_*_ values were obtained from Reynolds stress as ${u}_{*}=\sqrt{-\overline{u^{\prime}w^{\prime}}}$, with $u^{\prime}$ representing longitudinal flow fluctuations (Reidenbach et al. 2006; Inoue et al. 2011). In the current study, a 8-min window size was optimal and ensured the inclusion of major turbulent contributions while minimizing contribution from non-turbulent processes. The obtained O_2_ benthic fluxes were screened for data quality by (i) removing spikes due to sensor collisions with particles and debris, and (ii) flagging of measurements during abrupt flow direction changes. The screened vertical O_2_ fluxes, defined as negative for O_2_ uptake (or consumption) by the benthic community and positive for O_2_ production, were averaged to 2 h intervals for visualization and further analysis.

AEC measurements integrate an area of the seafloor upstream of the AEC position, which is termed the AEC footprint and defined as area of the seafloor that contributes to 90% of the flux (Berg et al. 2007). The theoretical dimensions of the footprint were estimated from *h* and the bottom surface roughness parameter (*z*_0_) following the parametrization of Berg et al. (2007). The surface area is expected to increase with increasing *h* and decrease with increasing bottom roughness. Values for *z*_0_ was quantified as ${z}_{0}=h\cdot {\exp}\left(-\kappa \cdot \frac{U}{{u}_{*}}\right)$ with $\kappa$ being the von Karman constant (0.41), and U the flow velocity magnitude (Wüest and Lorke 2003). As each flow direction is associated with a specific footprint, a particle track analysis was performed on the flow velocity data to identify consecutive periods of well-developed directional flow throughout the deployment duration, and thus to characterize the associated footprints. Dominant benthic communities and the relative habitat coverage by algae, benthic fauna, and substrate were obtained for each site-specific footprint by cross-referencing AEC sites and main flow directions with the previously obtained benthic images. Site-specific footprint areas were obtained by averaging areal contributions from each direction.

#### Benthic metabolism

Benthic respiration rates (ER, in mmol O_2_ m^−2^ d^−1^) were quantified from the mean nighttime benthic O_2_ uptake rate scaled to 24 h. Nighttime was defined as periods with PAR ≤ 0.2 µmol m^−2^ s^−1^. Net benthic production rates (NEP) were obtained as the mean of daytime (PAR > 0.2 µmol m^−2^ s^−1^) flux measurements. Consequently, benthic gross primary production (GPP, in mmol O_2_ m^−2^ d^−1^) was calculated as GPP = NEP + |ER| assuming a light-independent respiration rate (Lovett et al. 2006). Since daytime respiration in aquatic systems is generally higher than at night (see Fenchel and Glud 2000), GPP rates represent conservative estimates. The net ecosystem metabolism over 24 h (NEM, mmol O_2_ m^−2^ d^−1^) was quantified as NEM = NEP—|ER|. Positive NEM rates indicate net autotrophy, while negative values reflect net heterotrophic conditions.

## Results

### Metadata

**Fig. 2:**
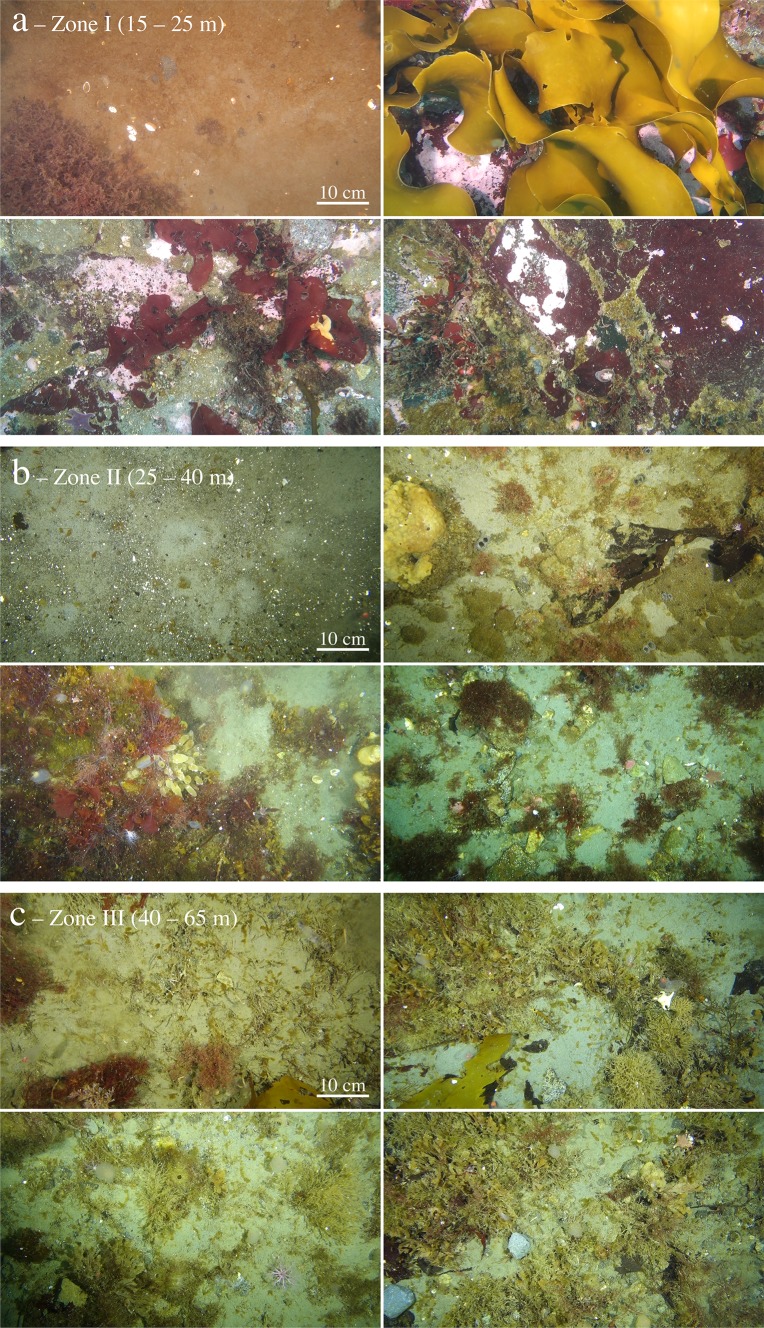
Mosaic of dominant benthic habitats observed during the benthic imaging transects within depth zone I (**a**), zone II (**b**), and zone III (**c**). Note that each image covers an area of 41 × 73 cm (0.3 m^2^)

South Bay was sea ice-free during the study period, with occasional presence of icebergs which drifted into the bay and at times inducing local benthic scouring before getting grounded in the shallows, especially in the South-West coast of the bay. Water temperature near the seafloor (i.e., 1 m above) ranged, on average, from 2.3 °C at the shallowest site (15 m depth) to 1.3 °C at the deepest site (58 m) with dampened tidal-driven diel variability (Supporting Information Table 1). The reported range was in line with the temperatures observed for the ice-free summer period of both 2016 and 2017 (Cárdenas et al. 2018a). The salinity at the targeted depth range remained constant throughout the sampling period at 32–33 PSU depending on depth. The O_2_ level in bottom waters was close to air-saturation at depths down to ~ 20 m (94 ± 5%) but undersaturated at the deeper sites (Online Resource 1), with a minimum value of 80 ± 2% at ~ 60 m depth. Measured benthic PAR was as high as 133 µmol quanta m^−2^ s^−1^ at 18 m, and up to 13 µmol quanta m^−2^ s^−1^ at 35 m depth. Daily-integrated PAR (PAR_24_) measured at the seabed decreased near-exponentially with increasing water depth, ranging from 1.7 mol quanta m^−2^ d^−1^ at the shallowest sites to ~ 0.1 mol quanta m^−2^ d^−1^ at 35 m depth, to values below detection at 59 m (Table 1). Mean near-seafloor flow velocity was characterized by complex flow patterns, which reflected the structural complexity of the benthic habitats. But generally, the flow velocity magnitude ranged from 1 to 4 cm s^−1^ with a mean flow direction going from south-west to north-east, i.e., into the bay (Online Resource 1). The mean bottom roughness length scale (*z*_0_) for all depths was ~ 5 cm. For the data presentation and discussion, the investigated depth range will be divided into zones: zone I (15–25 m depth range), zone II (25–40 m), and zone III (40–65 m) (Fig. 2).

**Table 1:** Overview of aquatic eddy covariance (AEC) deployments, benthic O_2_ fluxes, and resulting key metabolic rates

Zone—depth (m)	AEC	Duration (h)	Benthic O_2_ flux (mmol m^−2^ h^−1^)		Light period (h)	PAR_24_ (mol m^−2^ d^−1^)	GPP (mmol m^−2^ d^−1^)	ER (mmol m^−2^ d^−1^)	NEM (mmol m^−2^ d^−1^)
	Depl.		Light	Dark					
I—15	15	3.8^a^	0.12 ± 0.74 (8)	− 2.93 ± 0.65 (4)	16.7	1.80	50.9	70.3	− 19.4
I—15	17	36	− 0.66 ± 1.00 (11)	− 3.11 ± 0.55 (5)	16.5	1.50	40.4	74.7	− 34.3
I—15	18	10	0.49 ± 5.75 (2)	− 3.70 ± 5.11 (3)	16.7	1.80	70.1	88.9	− 18.8
I—20	16	20	− 0.61 ± 0.10 (19)			1.80		14.6	− 14.6
II—31	10	40	− 0.39 ± 0.08 (19)			0.02		9.4	− 9.4
II—31	12	78	− 0.58 ± 0.07 (61)			0.01		13.9	− 13.9
II—33	8	54	− 0.30 ± 0.05 (45)			0.14		7.1	− 7.1
II—35	6	34	− 1.48 ± 0.19 (19)	− 1.51 ± 0.16 (14)	16.5	0.07	0.6	36.3	− 35.8
II—36	1	66	− 0.14 ± 0.02 (41)	− 0.24 ± 0.05 (16)	16.6	0.07	1.5	5.8	− 4.0
II—36	5	58	− 0.43 ± 0.04 (52)			0.06		10.4	− 10.4
III—53	11	20	− 0.19 ± 0.05 (9)			0		4.6	− 4.6
III—58	13	46	− 0.06 ± 0.02 (17)			0		1.4	− 1.4
III—58	14	80	− 0.27 ± 0.04 (69)			0		6.4	− 6.4

Light and dark benthic fluxes are reported as whole-deployment mean values ± SE (n), with n representing the number of (2 h) averaged values. Benthic light availability is reported as daily-integrated PAR (PAR_24_, in mol quanta m^−2^ d^−1^). Representative benthic habitats for each AEC deployment, i.e., reference transects, were determined based on distance between AEC and imaging transect and flow directions, i.e., for the location of the theoretical AEC footprints. Note that AEC deployments 2–4, 7, and 9 were unsuccessful (AEC frame flipped-over due to the uneven bottom surface) and therefore discarded from further analyses

*GPP* gross primary, *ER* ecosystem respiration, *NEM* net ecosystem metabolism

^a^Sensor breakage after 4 h of deployment

### Biodiversity

**Fig. 3:**
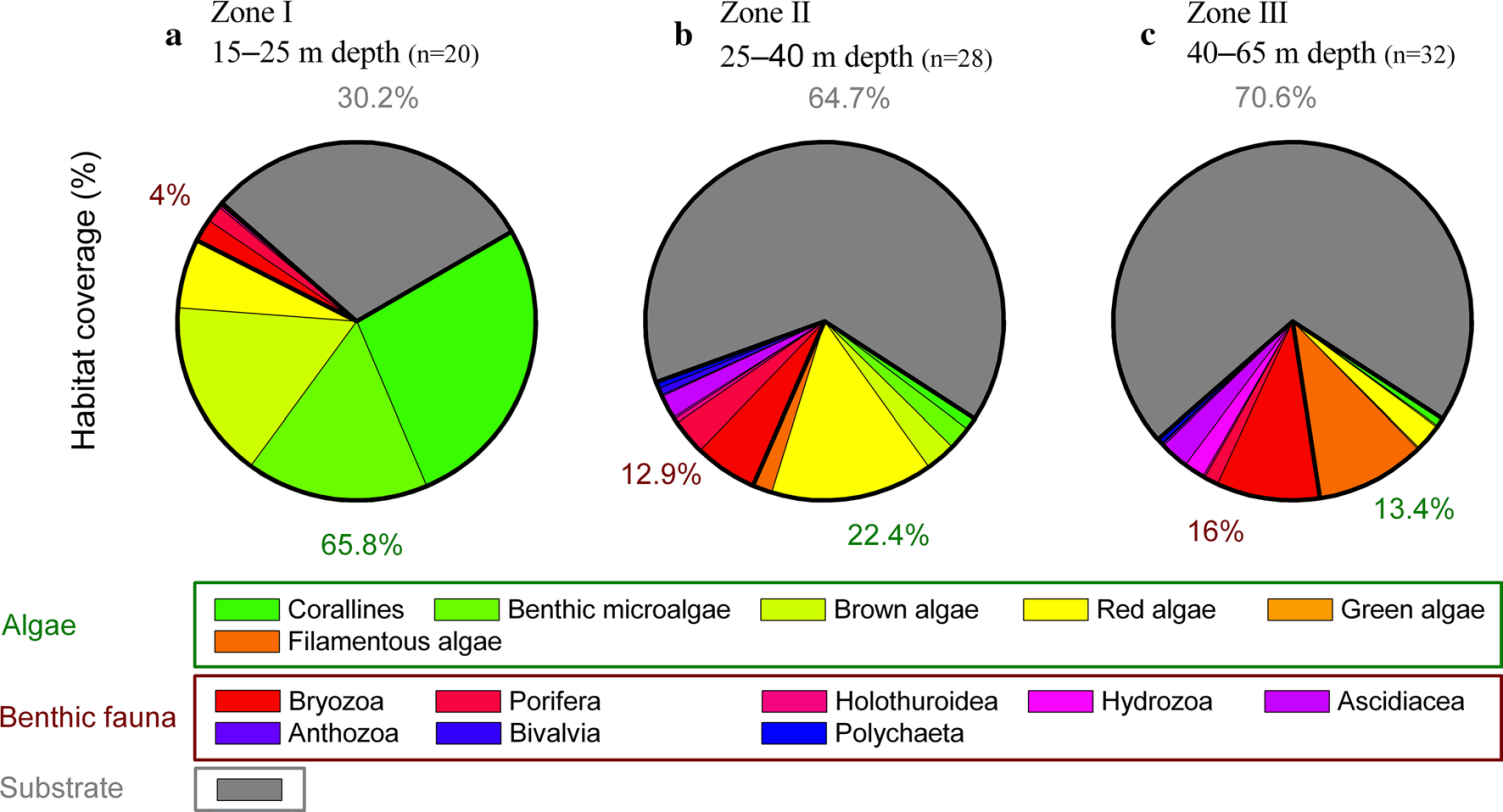
Summary of benthic habitat coverage by algae, fauna, and exposed (non-macrobiota-colonized) bare rocky substrate for zone I (depth range 15–25 m; **a**), zone II (range 25–40 m; **b**), and zone III (40–65 m; **c**). The number of images used for the visual characterization of each depth zone is reported under parenthesis. The colored sectors represent the relative coverage of each of the reported algae and benthic fauna groups, while the total habitat coverage by all algae and benthic fauna groups, as well as by bare rocky substrate is included as percentage. Rocky substrates were the dominantly exposed substrate type within the investigated imaging transects, with soft sediments only occurring as sparse surficial deposits. These were not separated from the rocky substrates in this study, though it should be noted that patchy soft-sediment habitats have been reported on the Eastern shore of South Bay within depths of 15–22 m (see Zamorano 1983)

**Fig. 4:**
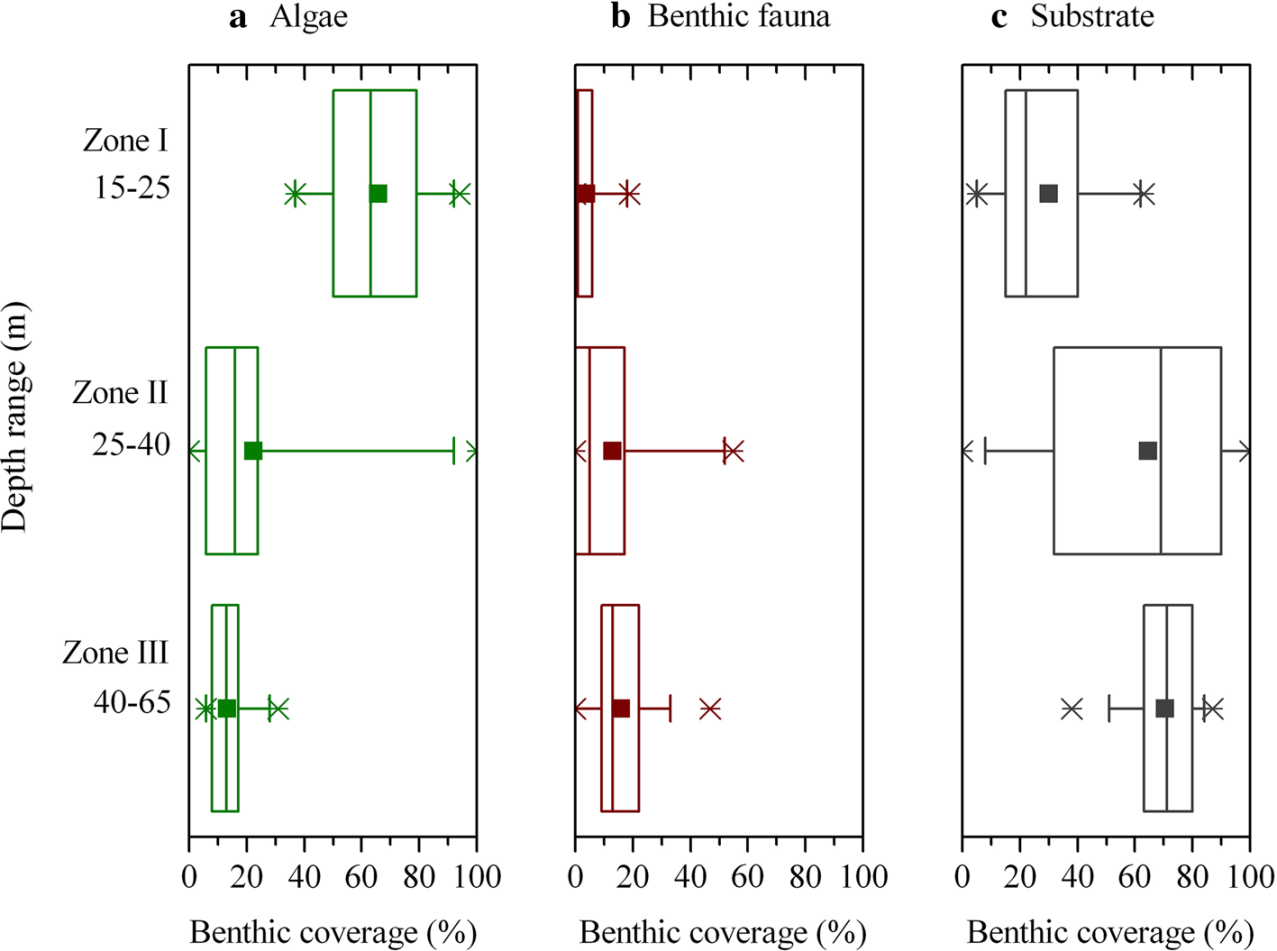
Variability of total benthic habitat coverage by algae (**a**) and benthic fauna (**b**) groups as well as by exposed rocky substrate (**c**) across the three depth zones. Each box plot shows the first, second (median), and third quartiles together with the mean value (solid square), with the whisker representing the 5% and 95% percentile, respectively. Cross symbols indicate the minimum and maximum values for each plot

Benthic imaging covered a depth range from 15 to 65 m and revealed a clear bathymetric change for both algal and faunal communities (Fig. 2). Within zone I, benthic habitats were dominated by algae. Most abundant, in percent of habitat coverage, were *H. grandifolius* and other brown macroalgae (32%) and encrusting corallines (27%), with foliose red algae making up the remaining 6%. Benthic fauna only covered 4% of the habitat and was dominated by bryozoans (52%) and sponges (38%) (Fig. 3). Bare rock showed high coverage values in zone I, reaching 30%. Benthic habitats in zone II were also characterized by the high coverage of bare rock, which represented on average 61% of the habitat coverage (Fig. 3). Coverage by algae was reduced to 22%, with red algae and brown algae representing, on average, 15% and 3% of the habitats, respectively. Benthic fauna coverage increased to 13% and was dominated by filter feeders, such as bryozoans (44%), sponges (25%), and ascidians (16%). Habitats in zone III were also largely dominated by rocky substrate (64%). Algal coverage was further reduced to 13%, with brown algae representing the majority of the algal community (10%), and red algae covering 2.4%. Benthic fauna represented 16% of the habitat coverage and was dominated by bryozoans (58%), with ascidians (16%), hydrozoans (12%), and sponges (8%) making up the remaining benthic coverage. The spatial distribution of both algae and benthic fauna was highly variable within each depth zone (Fig. 2). The largest variability was observed within zone II (Fig. 4) where the habitat topography was most complex, ranging from rocky patches to flat ice-scoured areas with transition zones from large rocks to pools of fine gravel (Fig. 2).

### Benthic fluxes

**Fig. 5:**
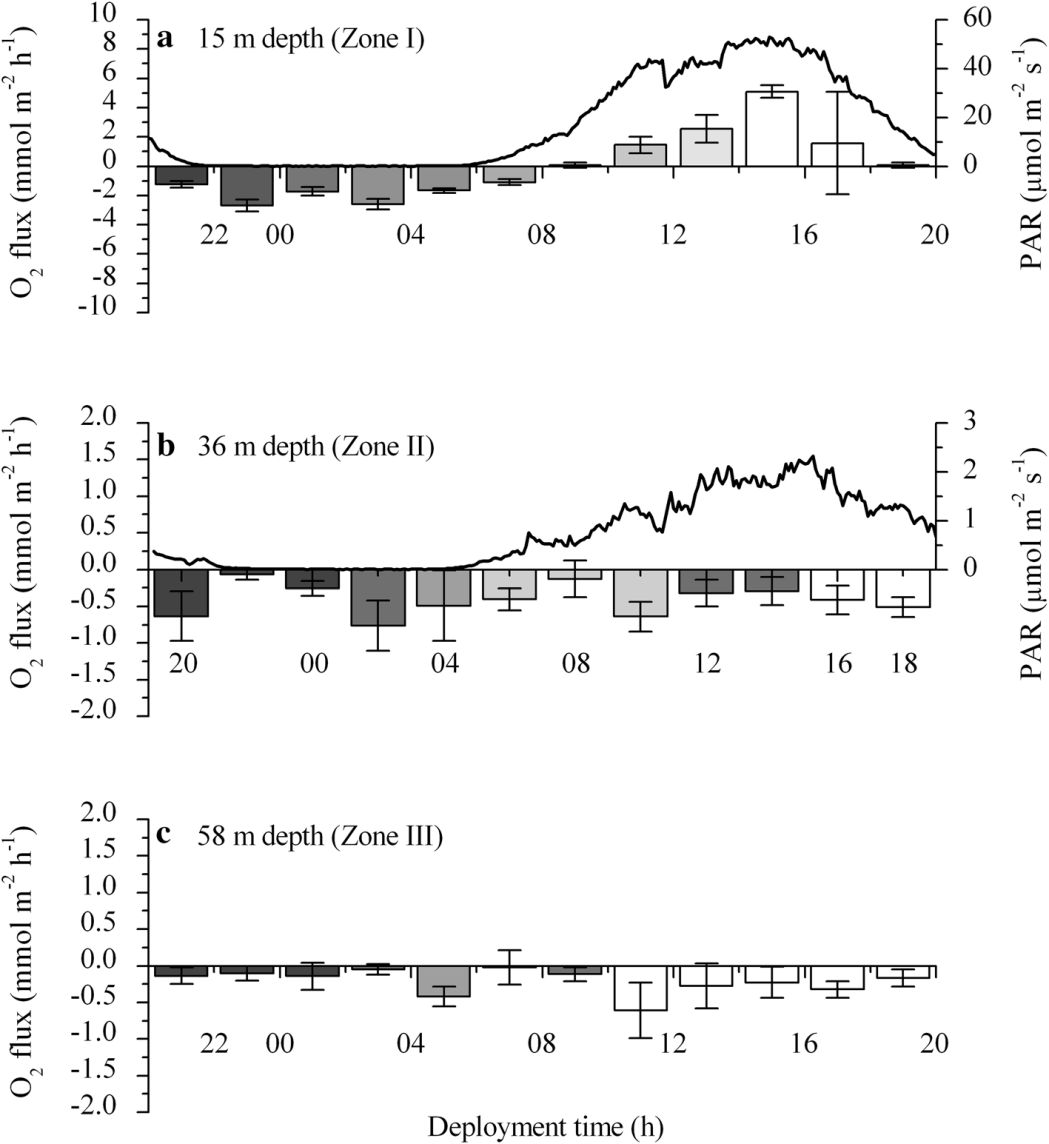
Typical 24-h time series of AEC-based benthic O_2_ fluxes and concomitant near-seabed photosynthetically active radiation (PAR) for zone I (**a** AEC17), zone II (**b** AEC05), and zone III (**c** AEC14). Each bar depicts the mean flux over 2 h and standard error. The dominant AEC footprint area integrated within each 2-hour flux was determined using a particle track analysis on the ADV flow measurements. Each bar color indicates a distinct footprint area, which is associated with consecutive shifts in the dominant flow direction from the start of each deployment. Matching colors are indicative of the same dominant flow direction

The in situ O_2_ fluxes along the depth transect were quantified from a total of 545 h of measurements. Typical 24-h time series for the three depth zones are illustrated in Fig. 5. Zone I had clear daily dynamics in O_2_ fluxes, which reflected the near-seabed light availability. Fluxes were negative at night and turned positive, or less negative, during the daytime (Fig. 5). Overall, hourly O_2_ fluxes (2-hours bins) ranged from − 6.56 to 6.25 mmol m^−2^ h^−1^, with mean daytime averages of − 0.66 to 0.49 mmol m^−2^ h^−1^ (Table 1). Mean O_2_ fluxes at night ranged between − 0.61 and − 3.70 mmol m^−2^ h^−1^. Zone I communities were well adapted to the light conditions and required as little as 4 µmol quanta m^−2^ s^−1^ to balance (i.e., compensate) community respiration with benthic primary production. Based on linear flux–PAR relationships, PAR dynamics were found to account for up to 60–90% of the observed variability in hourly averaged O_2_ fluxes. Within zone II, benthic O_2_ fluxes were mostly negative and showed a reduced diel dynamics and dampened response to light availably (Fig. 5). The O_2_ fluxes ranged from − 3.24 to 0.27 mmol m^−2^ h^−1^, with mean fluxes in the light and in the dark, ranging from − 0.14 to − 1.51 mmol m^−2^ h^−1^ across sites (Table 1). At the deeper aphotic sites, within zone III no daily dynamics were observed. These sites had persistent negative O_2_ fluxes, reaching up to − 1.46 mmol m^−2^ h^−1^ (2-hours bins). Mean daily fluxes ranged from − 0.06 to − 0.27 mmol m^−2^ h^−1^ (Table 1).

The O_2_ fluxes obtained at each site integrated a theoretical footprint area ranging from 5 to 25 m^2^ depending on the structural complexity of the benthic habitats. Due to shifts in the flow direction during each deployment, the resulting O_2_ flux time series included both temporal dynamics, i.e., light response, and spatial contributions from multiple footprints (Fig. 5). This provided a better integration of the spatial heterogeneity and patchiness of each site but impeded a meaningful temporal integration of light relationships, e.g., production-irradiance parametrizations, across the depth zones as these would be limited to O_2_ fluxes from matching footprints at each site.

Daily GPP quantified from light and dark O_2_ fluxes ranged from 40–70 mmol m^−2^ d^−1^ (average 54 mmol m^−2^ d^−1^) at zone I sites to < 2 mmol m^−2^ d^−1^ at zone II sites (Table 1) and 0 mmol m^−2^ d^−1^ within zone III. Daily ER for individual deployments ranged from a maximum of ~ 90 mmol m^−2^ d^−1^ within zone I to a minimum of 1.4 mmol m^−2^ d^−1^ at the deepest site. Mean depth-averaged ER rates decreased from 62.1 mmol m^−2^ d^−1^ at zone I sites, to 13.8 mmol m^−2^ d^−1^ at zone II, and 4.1 mmol m^−2^ d^−1^ at zone III (Table 1). All sites were characterized by a negative NEM throughout the observational period reflecting net heterotrophic conditions. Mean site-specific NEM rates ranged from − 1.4 to − 35.8 mmol m^−2^ d^−1^ (Table 1). NEM also showed a marked depth relationship, with mean zone rates decreasing in magnitude from − 21.8 mmol m^−2^ d^−1^ (zone I) to − 4.1 mmol m^−2^ d^−1^ (zone III).

## Discussion

### Benthic heterogeneity

**Fig. 6:**
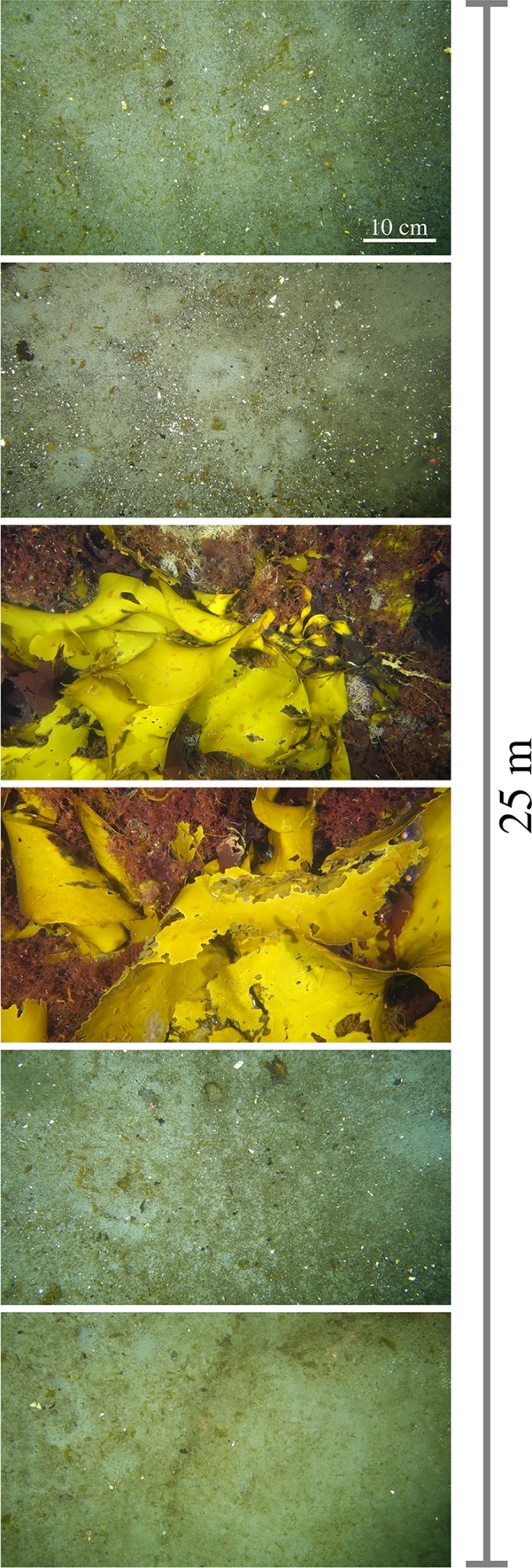
Example of the effect of ice scouring on benthic communities in South Bay (zone II, 35 m depth). Note that the imaging transect covers a length of 25 m, with each image representing a benthic area of 0.3 m^2^ (41 × 73 cm). Note that the exposed sand areas visible in the benthic images were indicative of a rocky substrate covered by a surficial deposit of sediment

In this study, benthic imaging and assessments of biodiversity were used to describe habitat heterogeneity, community structure, and highlight zonal trends of the hard-substrate benthic communities. As indicated by previous surveys in (shallow) Antarctic coastal settings (e.g., Nonato et al. 2000; Barnes and Brockington 2003; Bowden 2005; Lagger et al. 2018), the habitats within South Bay were characterized by continuous change along depths and high heterogeneity in some areas (Figs. 2, 3). Habitats down to about 40 m depth (zone I–II) appeared to be strongly affected by ice scouring. The imaging transects revealed the occurrence of a marked patchiness in the distribution of communities in those areas, with shifts between the presence and absence of well-established benthic macroalgae and epifauna, i.e., scoured and non-scoured areas, separated by only a few meters (Fig. 6). These observations are consistent with multiannual monitoring of ice disturbances carried out in other areas such as Adelaide Island, WAP (Smale 2008), where habitat shifts often occur on spatial scales down to 15 m between scoured and unaffected neighboring areas.

Zamorano et al. (1986) observed that faunal distribution in South Bay exhibits a marked shift from motile fauna, e.g., Antarctic limpets, at shallower areas (< 10 m), to large sessile benthos, e.g., ascidians and sponges, at depths down to 30 m. Such distribution was found to be consistent with the imprint of ice scouring on benthic communities, which favors motile benthic fauna (see Nonato et al. 2000; Bowden 2005). Although there was a change in the dominance of macroalgae and invertebrates between shallow and deeper zones, each zone area was highly heterogeneous. This study provides further evidence for the occurrence and effects of ice disturbances in South Bay. Image-based faunal distribution showed a marked increase in the abundance of small and large sessile fauna with increasing water depth (Figs. 2, 3), in agreement with an expected decrease in ice scouring events with increasing depth (Bowden 2005; Smale et al. 2008).

The combination of biodiversity assessments with AEC measurements employed herein enabled a more refined description of the habitat variability at each site and trends of depth zones within South Bay. For instance, benthic images confirmed an increase in the abundance of benthic fauna with depth, from 30 to 65 m (deepest AEC deployment site). AEC-based O_2_ fluxes were found to be highly variable, reflecting the observed heterogeneity and patchiness in benthic habitats (Fig. 5, Table 1). By integrating (i) contributions from mixed heterogenous communities, and (ii) contributions from multiple footprints according to the dominant flow directions, the obtained fluxes provided a robust representation of the benthic community activity between sites and across the respective depth zones.

### Ecosystem functioning

This study was performed during the peak of pelagic primary production in the Palmer Archipelago, which coincides with the ice-free period, from early Dec to Mar (Moline and Prézelin 1996; Goldman et al. 2015; Höfer et al. 2019). During this period, algal blooms, lasting up to 3 weeks, might account for up to half of the total pelagic production during the ice-free season (Goldman et al. 2015). Mean pelagic GPP rates in the area generally range from 1.08 to 6.58 g C m^−2^ d^−1^ during blooms (see Moline and Prézelin 1996), while peak pelagic GPP rates for South Bay might be as high as 14.46 g C m^−2^ d^−1^ (Höfer et al. 2019). The obtained rates of benthic GPP at South Bay (Table 1) were comparable in magnitude to those reported for Antarctic habitats on soft sediment during the ice-free period (e.g., Gilbert 1991; McMinn et al. 2010; Shim et al. 2011). However, these benthic GPP rates only compared to the lower end of the reported pelagic GPP range, with pelagic blooms exceeding benthic GPP by, on average, a factor of 20 (assuming a 1:1 carbon-to-O_2_ molar ratio).

All benthic habitats within each of the three investigated depth zones were net heterotrophic, i.e., negative NEM rates (Table 1), indicating that benthic ER was in excess of benthic GPP and that the habitats are sustained by an additional allochthonous supply of organic matter. In contrast to soft-sediment habitats, where the sediment plays a dominant role for both organic matter degradation and recycling of nutrients (see Middelburg et al. 2005), these rocky habitats rely on the community’s ability to draw down organic matter from the surrounding water. This was reflected by the habitats’ faunal community, which was dominated by suspension feeders, e.g., bivalves, bryozoans, ascidians, and sponges (Fig. 3). These organisms actively enhance the supply of organic material to the benthic community by filtering large volumes of water. For instance, Antarctic sponges such as *M. acerata* can typically filter up to 180 mL h^−1^ g^−1^ dry wt, while ascidians might filter up to 250–349 mL h^−1^ g^−1^ dry wt (Kowalke 1999, 2000). In addition, suspension feeders can also provide nutrition for other benthic organisms via feces, pseudofeces, or detritus production (e.g., Norkko et al. 2001; de Goeij et al. 2013). Tropical reef sponges, for example, have been shown to make dissolved organic matter available to other benthic organisms (de Goeij et al. 2013). However, this has not yet been investigated in Polar ecosystems.

### Carbon cycling by Polar habitats

**Fig. 7:**
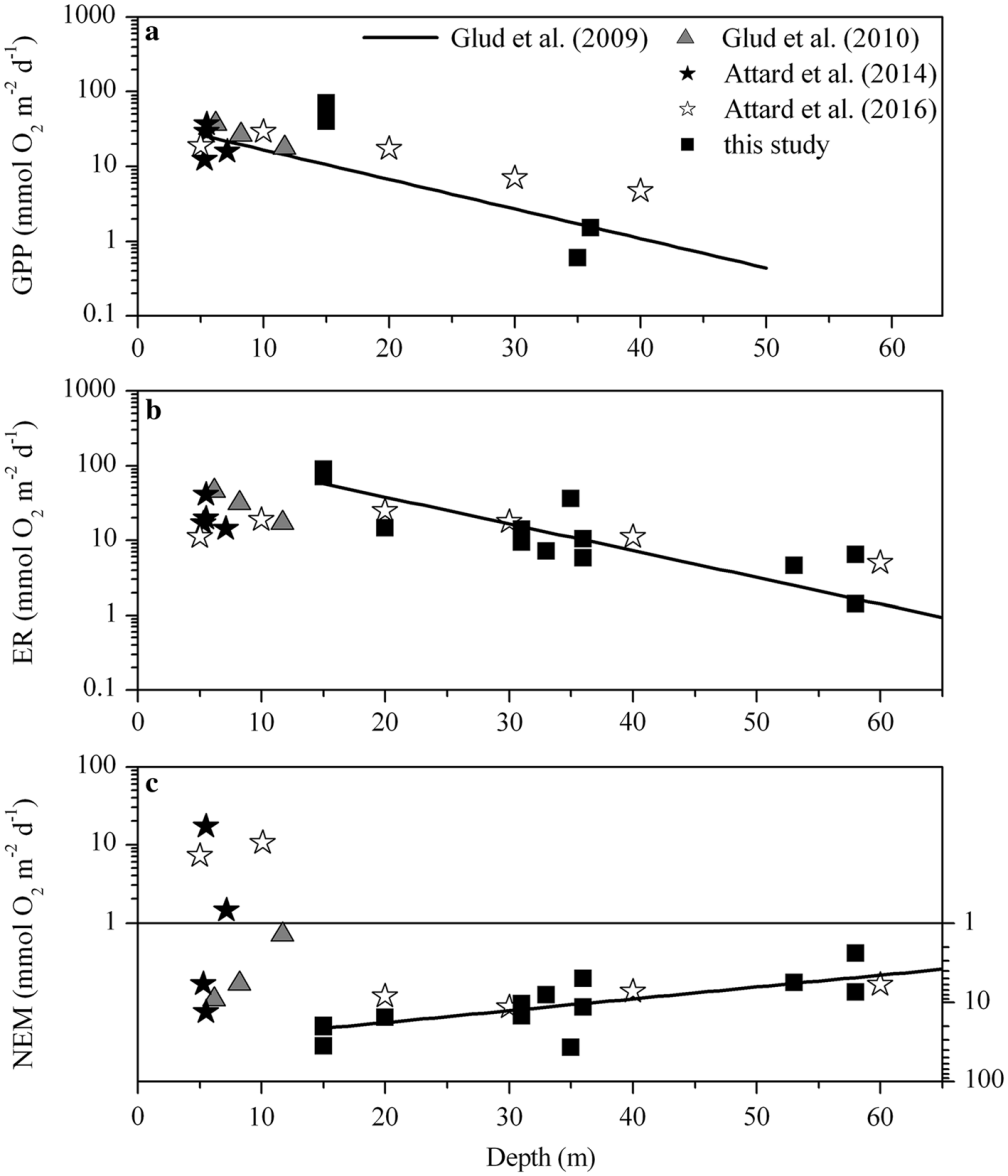
Depth relationship of gross primary production (GPP), ecosystem respiration (ER), and net ecosystem metabolism (NEM) for Polar benthic communities on hard substrates. **a** Rates of GPP (in mmol O_2_ m^−2^ d^−1^) from Arctic habitats are depicted from Glud et al. (2010), gray triangles, Attard et al. (2014), black stars, and Attard et al. (2016), white stars, respectively. Rates for South Bay are presented as individual sites (black squares). The trendline $GPP=41.5{e}^{-0.0911depth}$ (*R*^2^ = 0.41) was obtained from Glud et al. (2009) and is based on benthic microalgal production rates on soft sediments in the Arctic. **b** Rates of ER (in mmol O_2_ m^−2^ d^−1^) as a function of water depth for the same studies reported for **a**. Trendline $ER=30.1{e}^{-0.0082depth+1.8764}$ (*R*^2^ = 0.62), covers the depth range 15–65 m (see zones I–III in this study). **c** Depth relationship for NEM (in mmol O_2_ m^−2^ d^−1^) for the above studies, $NEM=35.8{e}^{-0.0345depth}$ (*R*^2^ = 0.35). Note that all values were offset by 1 mmol O_2_ m^−2^ d^−1^ to facilitate the visualization on a log-scale. Data below the line are negative indicating net heterotrophic conditions

Hard bottom communities dominate the coastal zones of many Polar regions, and the GPP of the present summer study from Antarctica compare well to values obtained for hard substrates at similar depths and light levels in the Arctic (e.g., Glud et al. 2010; Attard et al. 2014, 2016). In addition, the proposed depth relationship of GPP for Arctic habitats on soft sediment by Glud et al. (2009), which indicated a near-exponential decline in GPP with depth, was found to describe the activity encountered on hard substrates on both Arctic and Antarctic settings (Fig. 7a). This apparent depth relation of GPP in Polar regions would at first glance suggest that light availability determines GPP rates across the different benthic habitats. Although light availability has been indicated as one of the main drivers of local benthic GPP in Polar hard substrates (e.g., Glud et al. 2010; Attard et al. 2016), the generalized relation presented in Fig. 7a is in fact also modulated by (i) ice scouring and (ii) the depth distribution and relative abundance of suspension feeders.

Habitats < 15 m depth in Polar regions are strongly affected by ice scouring (e.g., Barnes 2017), which represents the dominant driver of the observed patchiness in the distribution of benthic algae and fauna, and of the resulting changes with depth (see discussion above). The reported GPP rates at those depths are typically lower than those for slightly deeper (~ 15 m), less scoured habitats, despite higher light availability (e.g., Attard et al. 2016) (Fig. 7a). The effect of ice scouring is also evident for the integrated ER rates (Fig. 7b). While ER rates for habitats > 15 m depth followed a near-exponential decrease with depth, ER rates for shallower sites were mostly clustered together and showed little relation with water depth. This is likely a result of seasonal ice disturbances, e.g., ice grounding and scouring, that inhibit the accumulation of faunal biomass and fine-grained organic material (e.g., Zamorano 1983; Attard et al. 2016), translating into lower ER rates than at the slightly deeper habitats (Fig. 7b).

During the summer time, NEM rates from available Polar habitats with little sediment deposition at depth > 15 m depth appeared consistently negative (Fig. 7c), indicating that active drawdown of organic material from the water column by suspension feeders is a key process in the biogeochemical and ecological functioning of high-latitude coastal ecosystems. NEM rates also decreased in magnitude with increasing water depth following a power function similar to the general global trend observed for the total O_2_ uptake rate in soft-sediment habitats (see Glud 2008). Shallower habitats (< 15 m depth) were characterized by a wide range of NEM rates, going from net heterotrophy (− 12.2 mmol m^−2^ d^−1^) to net autotrophy (to 16.2 mmol m^−2^ d^−1^). Such inherent variability presumably reflects the scouring of benthic habitats by ice at these depths, which strongly affects the occurrence and development of the algal communities and the abundance of benthic fauna.

This study, combined with recent assessment of the carbon turnover by rocky habitats in Arctic settings, contributes to shed light on the role of hard-substrate coastal communities in Polar regions. At present, there are still few measurements of metabolic rates for such habitats, despite their evident predominance. The introduction of flux approaches such as the AEC technique has provided a new tool for quantification of benthic metabolic rates in hard-substrate coastal habitats in both Arctic and Antarctic settings, and thus represents a further step toward more comprehensive characterizations of the carbon turnover by hard-substrate habitats in Polar regions.

## Electronic supplementary material

Below is the link to the electronic supplementary material.

Supplementary file1 (PDF 68 kb)
