# Supplementary material for: Benthic primary production and respiration of shallow rocky habitats: a case study from South Bay (Doumer Island, Western Antarctic Peninsula)
Source: Polar Biol. 2019 Jul 16;42(8):1459–74. doi: 10.1007/s00300-019-02533-0 (PMC6690856; doi:10.1007/s00300-019-02533-0)
Supplement: Supplementary file 1 — Supplementary file1 (PDF 68 kb) [file 300_2019_2533_MOESM1_ESM.pdf]

ELECTRONIC SUPPLEMENTARY MATERIAL

Benthic primary production and respiration of shallow rocky habitats: a case study from South Bay (Doumer Island, Western Antarctic Peninsula)

Lorenzo Rovelli[1,5*], Karl M. Attard[1,2], César A. Cárdenas[3], Ronnie N. Glud[1,4]

[1]Nordcee, Department of Biology, University of Southern Denmark, 5230 Odense M, Denmark.
[2]Tvärminne Zoological Station, University of Helsinki, 10900 Hanko, Finland.
[3]Departamento Científico, Instituto Antártico Chileno, Punta Arenas 6200965, Chile.
[4]Department of Ocean and Environmental Sciences, Tokyo University of Marine Science and Technology, 108-8477 Tokyo, Japan.
[5] now at Institute for Environmental Sciences, University of Koblenz-Landau, Landau, Germany.
*Corresponding author: lorenzo@biology.sdu.dk

2 Tables

Table 1 Physicochemical characteristics at each deployment site. Values for the respective parameters are reported as mean ± standard deviation (SD) with minimum–maximum values presented within brackets.

Zone<br>Depth (m)	AEC<br>Depl.	Coordinates<br>Date (time)	Temperature<br>(°C)	O_2<br>(% sat)	O_2<br>(μmol L^{-1})	PAR<br>(μmol quanta m^{-2} s^{-1})
I –15	15	64° 52’ 13.84’’ S, 63° 34’ 25.61’’ W	2.0 ± 0.2	88.6 ± 2.6	306.7 ± 7.7	-
		14 Feb 2017 (19:00)	[1.7–2.4]	[83.4–93.7]	[290.3–321.6]	[0–133.3]
I –15	17	64° 52’ 28.09’’ S, 63° 34’ 36.01’’ W	2.3 ± 0.1	100.0 ± 2.2	343.5 ± 6.3	-
		17 Feb 2017 (20:00)	[2.2–2.6]	[93.5–108]	[328.4–368.8]	[0–52.9]
I – 15	18	64° 52’ 13.84’’ S, 63° 34’ 25.57’’ W	2.3 ± 0.01	92.7 ± 0.9	356.7 ± 3.1	-
		17 Feb 2017 (20:00)	[2.1–2.4]	[89.9–94.3]	[346.9–361.5]	[0–133.3]
I – 20	16	64° 52’ 15.28’’ S, 63° 34’ 4.91’’ W	1.9 ± 0.1	94.4 ± 2.5	328.5 ± 7.9	-
		15 Feb 2017 (21:00)	[1.6–2.1]	[88.6–100.2]	[310.0–347.2]	[0–133.3]
II – 31	10	64° 52’ 20.93’’ S, 63° 34’ 24.89’’ W	2.0 ± 0.2	84.8 ± 2.6	293.2 ± 7.9	-
		4 Feb 2017 (21:00)	[1.7–2.4]	[79.8–92.2]	[277.8–316.6]	[0–1.4]
II –31	12	64° 52’ 21.58’’ S, 63° 34’ 26.69’’ W	1.9 ± 0.2	83.5 ± 3.7	361.1 ± 14.4	-
		7 Feb 2017 (10:00)	[1.6–2.5]	[77.3–94.8]	[337.5–405.9]	[0–3.5]
II – 33	8	64° 52’ 20.50’’ S, 63° 34’ 27.91’’ W	2.0 ± 0.3	85.0 ± 4.2	294.2 ± 12.5	-
		1 Feb 2017 (20:00)	[1.6–2.7]	[78.3–94.4]	[273.8–321.9]	[0–8.1]
II – 35	6	64° 52° 19.49’’ S, 63° 34’ 24.38’’ W	2.1 ± 0.3	86.8 ± 3.2	299.1 ± 9.5	-
		29 Jan 2017 (21:00)	[1.5–2.5]	[78.8–91.9]	[275.9–315]	[0–5.0]
II – 36	1	64° 52’ 18.26’’ S, 63° 34’ 25.82’’ W	0.6 ± 0.08	74.6 ± 3.2	265.4 ± 10.6	-
		19 Jan 2017 (19:00)	[0.5–0.86]	[67.8–82.2]	[242.8–290.9]	[0–13.3]
II – 36	5	64° 52’ 18.55’’ S, 63° 34’ 25.79’’ W	1.8 ± 0.3	85.8 ± 3.2	297.5 ± 9.3	-
		24 Jan 2017 (19:00)	[1.0–2.2]	[77.5–92.2]	[273.8–318.4]	[0–2.9]
III – 53	11	64° 52’ 18.05’’ S, 63° 34’ 37.56’’ W	1.5 ± 0.1	82.5 ± 2.1	323.5 ± 7.5	-
		6 Feb 2017 (19:00)	[1.3–1.8]	[78.2–87.4]	[308.1–340.3]	[0]
III – 58	13	64° 52’ 18.05’’ S, 63° 34’ 38.68’’ W	1.3 ± 0.1	77.8 ± 2.2	306.9 ± 8.1	-
		8 Feb 2017 (10:00)	[1.1–1.7]	[72.5–82.5]	[287.8–323.4]	[0]
III – 58	14	64° 52’ 18.34’’ S, 63° 34’ 38.93’’ W	1.3 ± 0.1	79.4 ± 2.6	280.5 ± 8.5	-
		11 Feb 2017 (08:00)	[1.0–1.5]	[72.6–87.0]	[258.4–305.9]	[0]

Table 2 Descriptive statistics on the benthic habitat coverage by algae, benthic sessile fauna and substrate for each depth zone.

Parameter	Depth Zone	Benthic habitat coverage (%)						
		Mean	SD	SE	Min	Max	Range	n
Algae	I	65.8	18.0	4.0	37	94	57	20
	II	22.4	24.3	4.6	0	100	100	28
	II	13.4	6.4	1.1	6	31	25	32
Fauna	I	3.9	5.9	1.3	0	19	19	20
	II	12.9	16.3	3.1	0	55	55	28
	II	16.0	10.5	1.9	0	47	47	32
Substrate	I	30.2	18.5	4.1	5	63	58	20
	II	64.7	29.5	5.6	0	100	100	28
	II	70.6	11.1	2.0	38	87	49	32
